# SRP-Net: sensitive risk propagation network with asymmetric cross-attention for educational data

**DOI:** 10.1038/s41598-026-53482-8

**Published:** 2026-06-04

**Authors:** Junpeng Hu, Xiao Guo, Tao Hu, Jinan Shen, Minghui Zheng

**Affiliations:** 1 College of Intelligent Systems Science and Engineering , Hubei Minzu University, Enshi, 445000 China; 2 School of Cyber Science and Engineering, Sichuan University, Chengdu, 610065 China

**Keywords:** Structured educational data, Sensitive field detection, Knowledge graph injection, Asymmetric cross-attention, Dynamic subgraph extraction, Low-resource learning, Computational biology and bioinformatics, Engineering, Mathematics and computing

## Abstract

In the digital transformation of education, detecting sensitive information in structured educational data faces the challenge of semantic mismatch between titles and content, as well as isolated risk assessment that ignores field relevance. We propose the Sensitive Risk Propagation Network (SRP-Net), which is a sequential two-stage architecture that aligns within the field and then propagates across fields. The first stage adopts asymmetric cross attention, treating field names as queries to align with instance values and statistical fingerprints (key values). In the second stage, these aligned representations are input into a sensitivity aware graph neural network through batch dynamic subgraph extraction to avoid excessive smoothing. We have developed Edu KG BERT for domain adaptation through structured knowledge injection and developed the open-source Edu-Sens, which is the first benchmark dataset for education sensitive data synthesized using privacy preserving protocols. SRP-Net achieved an accuracy of 0.8779 Macro-F1 and 95.58% accuracy on Edu-Sens, which is 3.06% higher than the non graph baseline and significantly better than traditional machine learning and pure semantic baselines. The ablation study confirmed the synergistic necessity of explicit in field alignment and dynamic cross field propagation.

**Supplementary Information:**

The online version contains supplementary material available at 10.1038/s41598-026-53482-8.

## Introduction

With the advancement of educational digital transformation, smart campuses, online teaching platforms, and educational government affairs systems have accumulated massive amounts of structured data. This production factor not only carries the core value of modernizing educational governance capabilities but also contains a large amount of highly sensitive information involving student and teacher privacy (e.g., ID numbers, family economic status, biometric features) and critical business confidentiality (e.g., research achievements, examination questions)^1^. In recent years, with the implementation of the *Data Security Law of the People’s Republic of China* and the *Guidelines for Educational Data Classification and Grading*^2,3^, establishing an accurate data classification and grading system has become the primary task of educational data security governance. In this compliance closed loop, sensitive field detection serves as the pre-requisite key technology for identifying data attributes and clarifying data asset inventories, and its accuracy directly determines the effectiveness of subsequent differential privacy protection and access control strategies.

However, in the real educational data ecosystem, achieving high-precision, automated sensitive field detection faces tremendous technical challenges. Unlike text sequences in natural language processing (NLP) that have rich contextual contexts, fields in relational databases often exhibit dual characteristics of semantic poverty and distributional heterogeneity^4^:

First Gap: Cross-modal Semantic Gap. In legacy systems or scenarios with non-unified development standards, field naming often adopts ambiguous abbreviations (e.g., “xh” referring to student ID), meaningless codes (e.g., “col_01”), or even mixed pinyin writing. Traditional rule-based matching or header-only models are prone to failure when facing such invisible features^5^, causing large amounts of sensitive data to become unregulated dark data.

More challenging is that when semantic conflicts exist between headers and instance values (e.g., a field named “political status” storing enrollment method information like “unified enrollment/direct admission”), single-channel models lack cross-modal verification mechanisms and struggle to make robust decisions^6,7^.

Second Gap: Isolated Risk Perception. Existing methods generally treat fields as independent samples for classification, ignoring the inherent structured correlation relationships in educational data^8^. For example, “ID number” and “bank account number” usually belong to the “student basic information” business domain, sharing similar sensitivity levels and access control strategies; while “student status” and “enrollment method” form semantic coupling through standard code references. Traditional multi-modal fusion (e.g., simple concatenation or gated weighting) can integrate multi-dimensional features within fields (headers, instance values, statistical fingerprints) but cannot propagate sensitive risk information between fields^9,10^, causing models to struggle to utilize structured prior knowledge in small annotated samples and resulting in insufficient generalization capabilities in low-resource scenarios^11^. Moreover, since full-graph propagation in Graph Neural Networks (GNNs) tends to produce over-smoothing problems when processing large-scale educational data^12^, the discrimination boundaries between high-sensitivity and low-sensitivity categories become homogenized, seriously weakening risk perception accuracy.

To address these challenges, we propose the Sensitive Risk Propagation Network (SRP-Net), an explicit multi-modal fusion and structured risk propagation framework for low-resource educational data scenarios. This framework constructs a dual-layer architecture of “within-field explicit alignment and cross-field risk propagation,” achieving field-level cross-modal fusion through asymmetric cross-attention and field-level risk propagation through dynamic subgraph extraction.

The main contributions of this paper are as follows:

1. We construct and open-source the first high-fidelity educational sensitive data test set (Edu-Sens). Addressing the common pain points in the data security field where real data is unavailable and public data is divorced from business, we collaborated with information centers from two domestic universities to collect real and complex business system data dictionaries, and innovatively employed a hybrid privacy-preserving synthesis technology driven by Large Language Models (LLMs) and rule engines to generate instance values. The dataset contains 3,462 real table headers and 2,942 training samples, reproducing the challenges of non-standard naming, cross-modal semantic conflicts, and severe class imbalance that exist in real industrial scenarios, filling the gap in publicly available validation sets for data classification and grading in the education field.
2. We propose a domain semantic injection mechanism based on an educational knowledge graph (Edu-KG-BERT). Addressing the common sense blind spots of general-purpose large language models in educational professional contexts, we constructed a triplet knowledge graph covering core educational nouns and business logic. Given the difficulty of obtaining large-scale open text for traditional pre-training in the education field, we designed an entity-level supervised knowledge injection method that explicitly models domain entity relationships and constructs hard negative samples, achieving efficient domain adaptation on small amounts of structured data and significantly lowering the data threshold for vertical domain model customization.
3. We design SRP-Net, an integrated architecture for explicit multi-modal alignment and risk propagation. An asymmetric cross-attention mechanism uses field names as Query and instance values/statistics as Key-Value, modeling the directional dependency “header → content.” A dynamic subgraph-based risk propagation strategy constructs sensitivity-aware graphs with business-domain and standard-code edges, achieving efficient message passing via batch-wise subgraph extraction to avoid full-graph over-smoothing.
4. Through controlled experiments, we establish the multi-modal fusion design paradigm of “structural prior superior to parameter expansion” for low-resource vertical domains. At the cross-field level, we confirm that dynamic subgraph extraction strategies significantly enhance discrimination boundary clarity while reducing computational complexity compared to full-graph propagation, effectively avoiding over-smoothing. At the within-field level, we validate that asymmetric cross-attention achieves explicit modal alignment through residual connections and layer normalization, improving Macro-F1 by 0.82% compared to Double-Gated fusion (0.8779 vs 0.8697) and by 1.41% compared to AGDN-Res (0.8779 vs 0.8638) with equivalent parameter scales. These findings provide empirical evidence for lightweight architecture design in educational data and similar scenarios.

## Related work

### Semantic type detection in tabular data

Semantic type detection in structured data is fundamental to data integration and knowledge discovery. Early methods mainly relied on manually defined rules and regular expressions (Regex), which required deep domain expertise and struggled to address the diversity and non-standardization of field naming. With the development of representation learning, researchers began exploring context-aware detection methods. Zhang et al.^13^ proposed the Sato framework, which introduced topic modeling (LDA) and table-level context information based on Sherlock, significantly improving semantic type recognition accuracy for open-domain tables. Subsequently, Deng et al.^14^ proposed TURL, which used masked entity prediction tasks for self-supervised pre-training on massive Web tables, proving the effectiveness of large-scale table pre-training models in cross-table transfer.

However, these methods face severe challenges in Chinese educational data domains. First, limited by historical legacy systems or manual entry habits, Chinese field names often exhibit arbitrary naming or severe semantic ambiguity (e.g., system-default generated columns like “backup column 1,” “extended field A,” or semantically inaccurate names like “holding telephone,” “current status”), causing models that only look at table headers to face severe semantic absence or misleading information. Second, existing general benchmark datasets mostly originate from open-domain Web tables, lacking deep representation of Chinese educational vertical business logic (e.g., poverty registration, unified/direct admission, political status codes)^15^. Therefore, relying solely on traditional statistical features or literal semantics of table headers is difficult to achieve high-precision sensitive information detection in real Chinese educational ecosystems.

### Domain pre-training and knowledge injection

Pre-trained Language Models (PLMs) have greatly improved baseline performance in NLP tasks through self-supervised learning on massive corpora. For structured data, Suhara et al.^6^ proved that using pre-trained models for serializing columns can effectively capture column-level context semantics, providing powerful foundational representations for table understanding. To address domain adaptation issues of general models, Gururangan et al.^16^ proposed the Domain-Adaptive Pre-Training (DAPT) paradigm, significantly improving models’ understanding of vertical industry terminology through continued learning on domain corpora. In terms of knowledge enhancement, Liu et al.^17^ proposed K-BERT, which injects triplet structures from knowledge graphs into pre-trained models, explicitly modeling entity relationships and effectively alleviating the common sense blind spots of general language models in professional domains.

However, in educational sensitive data detection scenarios, these methods still face challenges: general table pre-training models lack prior knowledge of sensitive entities in the education industry (e.g., student status codes, political status codes). Inspired by this, we construct an education-specific knowledge graph.

Recent advances extend knowledge-graph perception to hypercomplex relational structures. Li et al.^18^ propose Hypercomplex Graph Collaborative Filtering, which leverages Cayley–Dickson construction and graph convolution in hypercomplex space to capture high-order connectivity. This demonstrates that deep semantic coupling between entity relations and collaborative signals significantly improves representation quality, corroborating our motivation for structured knowledge injection in educational data.we therefore implement structured knowledge injection, aiming to endow models with deep understanding of entity relationships in the educational domain, thereby bridging the semantic gap caused by field naming.

### Multi-modal fusion for structured data

Sensitive field detection in structured data is essentially a multi-modal fusion problem, requiring coordinated processing of three heterogeneous signals: header text (metadata semantics), instance value sequences (content evidence), and statistical fingerprints (distribution features). Early studies mostly adopted feature-level simple concatenation or decision-level voting fusion^14^. Such methods flatten different modal features, forcing models to passively learn cross-modal alignment relationships in hidden spaces, lacking explicit constraints on dependencies between field names and value content. Therefore, when header semantics are fuzzy (e.g., “xh” referring to student ID) or cross-modal semantic conflicts occur (e.g., “political status” field storing enrollment methods), traditional methods lack cross-channel verification mechanisms and struggle to make robust decisions^20^.

In recent years, researchers have proposed complex deep fusion mechanisms to enhance representation interaction capabilities. Gated weighted fusion calculates adaptive weights for each modality through Sigmoid or Softmax, achieving fine-grained information screening^19,20^; Hierarchical Bi-directional Attention^21^ introduces multi-layer cross-attention to model high-order interactions between modalities; AGDN^22^ construct field attributes as heterogeneous graphs, utilizing multi-order neighborhood aggregation to propagate features. However, these methods expose structural adaptation deficiencies in typical low-resource vertical scenarios like educational data: First, multi-layer gating causes gradient competition and information filtering, causing models to fall into local optima^20^; Second, multi-order propagation in AGDN easily causes feature over-smoothing, making discrimination boundaries between high-sensitivity and low-sensitivity categories tend toward homogenization^22^; Third, over-parameterization caused by stacked parameters easily triggers overfitting on educational datasets with only thousands of samples^21^.

More critically, existing methods mostly adopt implicit fusion strategies, lacking explicit constraints on cross-modal alignment relationships, and lack error correction capabilities in the face of adversarial samples with semantic conflicts between headers and instance values. Beyond tabular semantic fusion, recent work addresses challenges that SRP-Net also faces: label noise and graph incompleteness^23,24^, signed relational structures^25^, and cascading risk propagation across interconnected nodes^26–28^. These studies validate the universality of higher-order neighborhood aggregation and graph-based risk quantification, yet their application to educational sensitive data remains unexplored. Through explicit Query-Key-Value structures—using field names as Query and instance values and statistical fingerprints as Key-Value—SRP-Net achieves direct attention from header metadata to heterogeneous information, effectively avoiding overfitting risks in low-resource scenarios while minimizing parameters, and significantly enhancing robustness when cross-channel semantic conflicts occur.

### Positioning of novelty

To clarify the architectural choices of SRP-Net against preceding paradigms, we explicitly contrast our method with three representative lines of work.

Knowledge injection. K-BERT^17^ inserts triplets as auxiliary tree branches into the Transformer input layer and employs a visible matrix to suppress knowledge noise. This is essentially an input-level augmentation that still relies on downstream fine-tuning to activate domain facts. By contrast, Edu-KG-BERT recasts knowledge injection as a standalone supervised triplet factuality classification task: the encoder is explicitly trained to discriminate valid educational triplets from hard negatives generated under relation constraints, after which the classification head is discarded and only the encoder weights—now deeply impregnated with domain priors—are preserved. This yields parameter-level knowledge solidification rather than input-level concatenation, which is more data-efficient in low-resource vertical domains.

Table understanding and multi-modal fusion. TURL^14^ pre-trains on massive Web tables via masked entity prediction for cross-table transfer, while Suhara et al.^6^ serialize columns into token sequences for semantic type detection. These methods excel at table-level or column-level context modeling, yet they do not natively support the field-level sensitivity attribution task, nor do they incorporate statistical fingerprints as an explicit modality. Moreover, their open-domain pre-training corpora lack the administrative codes and business logic specific to Chinese educational systems. SRP-Net bridges this gap by introducing an asymmetric cross-attention mechanism that treats the field name as Query and the instance value/statistical fingerprint as Key-Value, thereby modeling the directional dependency “header → content” and enabling robust correction when header semantics are noisy or misleading.

Graph-based risk propagation. AGDN^22^ and related heterogeneous GNNs perform full-graph propagation to aggregate multi-order neighborhood information. In the context of large-scale educational data, such global diffusion tends to over-smooth node representations and homogenize the discrimination boundaries between high- and low-sensitivity categories. SRP-Net instead adopts a batch-wise dynamic subgraph extraction strategy: a sensitivity-aware graph is constructed from training-set vocabulary, and only the induced subgraph of the current mini-batch is fed into a shallow 2-layer GAT. Edge weights are dynamically scaled by endpoint sensitivity differences, so that risk information propagates in a gradient manner rather than through uniform feature diffusion. This design explicitly trades full-graph coverage for local discriminative fidelity, which is critical when annotated samples are scarce.

## Problem definition and dataset construction

### Problem formulation

We define the educational data sensitive information detection task as a multi-modal feature-driven three-class classification problem. Given a database field C to be detected, its input features consist of three core dimensions:

- Header Metadata (H): The display name of the field and its associated annotations, e.g., “student mobile phone.”
- Instance Value Sequence (V): A text sequence composed of k real observations randomly sampled from the database: $V = \left\{ {v_{1} ,v_{2} , \ldots ,v_{k} } \right\}$.
- Statistical Fingerprint (S): A numerical feature vector extracted through explicit rules: $S \in R^{d}$, including mean, uniqueness rate, regular expression hit rate, etc.^29^.

The goal of the model is to learn a nonlinear mapping function f to predict its sensitivity level label y given the input modalities,as defined in formula 1:

1 $$y = f\left( {H,V,S;\theta } \right)$$

where the label space Y ∈ {L1,L2,L3}, corresponding to Public Level, Medium Sensitivity Level (Internal), and High Sensitivity Level (Extreme Privacy), respectively.

The complete notation and symbol definitions are provided in Supplementary Material Table 3.

### Edu-sens dataset construction

Since educational sensitive data is strictly protected by the Data Security Law, directly disclosing real production environment data poses compliance risks. Therefore, we collaborated with information centers from two Chinese universities to construct the Edu-Sens benchmark dataset through the paradigm of real headers combined with privacy-preserving synthesis.

#### Real-world schema collection

We exported complete data dictionaries from educational management systems (EMS), student affairs systems (SAIS), and campus card consumption systems from two universities. The collection process obtained 3,462 independent field names. These field names fully preserve the naming diversity in real business scenarios, as shown in Table 1:

**Table 1:** Examples of naming diversity in educational data fields.

Category	Field name examples	Field value examples
I. Explicit semantic	ID Number Bank Account Home Address	11010119900101xxxx 622202xxxxxx No. xx Road, Haidian District, Beijing
II. Fuzzy name—discernible value	Extended field A Backup column	2,021,001,001 13,800,138,000 Zhang San School of computer science
III. Statistical feature dependent	grade consumption amount library borrowing count	85.5 23.50 12
IV. Name-value misalignment	Political status Enrollment Method Remarks	Unified/direct/reduced score In progress/archived This student has family economic difficulties…

The above four categories reflect the complexity of real educational data governance. Particularly for Category IV (disruptors), which originate from field mapping errors during system migration or manual entry mistakes, traditional rule-based or single-column-name detection methods will inevitably fail in such scenarios, urgently requiring intelligent models with multi-channel cross-verification capabilities.

#### Privacy-preserving hybrid synthesis

To restore the statistical distribution of underlying data without leaking privacy, we designed a dual-engine synthesis scheme:

1. Rule-Driven Engine (Faker Engine): For fields with strong validation rules (e.g., ID cards, bank accounts, mobile phones), we adopt the Python Faker library combined with Chinese National Standards (GB/T) to generate simulation data. Such rule-based synthesis methods maintain the format validity of data while effectively preserving the statistical characteristics of original data^30^.
2. Generative AI Engine (LLM Engine): For weak-rule long-tail text fields (e.g., award speeches, disciplinary reasons, family background descriptions), we use Large Language Models (LLMs) to input field semantic contexts and generate highly semantically rich simulation samples. Recent studies show that LLMs demonstrate strong context-aware capabilities in domain-specific data augmentation and synthetic data generation tasks^31^.

#### Robustness noise injection

To simulate data quality issues caused by system migration or entry errors in real environments, we deliberately retained approximately 5% mismatch samples during construction. For example, mixing enrollment method information like “unified/reduced score” into a field named “political status.” This design aims to train models with robust performance to correct decisions through underlying instance values when header semantics fail. Label noise and data inconsistency are common problems in actual business systems, and moderate noise injection helps improve model generalization capabilities^32^.

#### Labeling system and data distribution

During labeling, three experts conducted back-to-back labeling of all fields referring to the Educational System Data Security Management Standards. Given the severe class imbalance in educational sensitive data (high-sensitivity samples account for only 4.19%, consistent with the scarcity of core privacy data in reality), we adopted category-weighted labeling strategies and expert verification mechanisms to alleviate the impact of class imbalance on model training^33^.

Finally, the dataset was divided into training set (2,942 samples) and test set (520 samples) in an 8.5:1.5 ratio. To promote industry research, Edu-Sens has been publicly released on the Kaggle platform (https://www.kaggle.com/datasets/baiputao/edu-sens).

To demarcate the boundary between real and synthetic components, we systematically quantified the provenance of Edu-Sens. All 3,462 field headers originate from the real-world data dictionaries of two partner universities (educational management systems, student affairs systems, and campus-card systems), yielding a 100% real ratio for schema metadata. After deduplication, null-value filtering, and compliance review, 3462 sample records with synthetic instance values were produced, so the instance-value synthetic ratio is 100%. Specifically, 2244 samples (64.9%) correspond to strong-validation-rule fields (e.g., ID numbers, bank accounts, mobile phones, student-status codes) generated by the Faker engine in compliance with GB/T national standards, while 1045 samples (30.2%) are weak-rule, long-tail text fields (e.g., award speeches, disciplinary reasons, family background descriptions, remarks) produced by the LLM engine. In addition, approximately 5% (173 records) of intentionally misaligned name–value pairs were retained to simulate real-world data-quality issues. This quantitative composition ensures that the dataset preserves authentic business naming diversity and structural associations while completely eliminating exposure of genuine personal information.

## Proposed methodology: SRP-Net framework

**Fig. 1:**
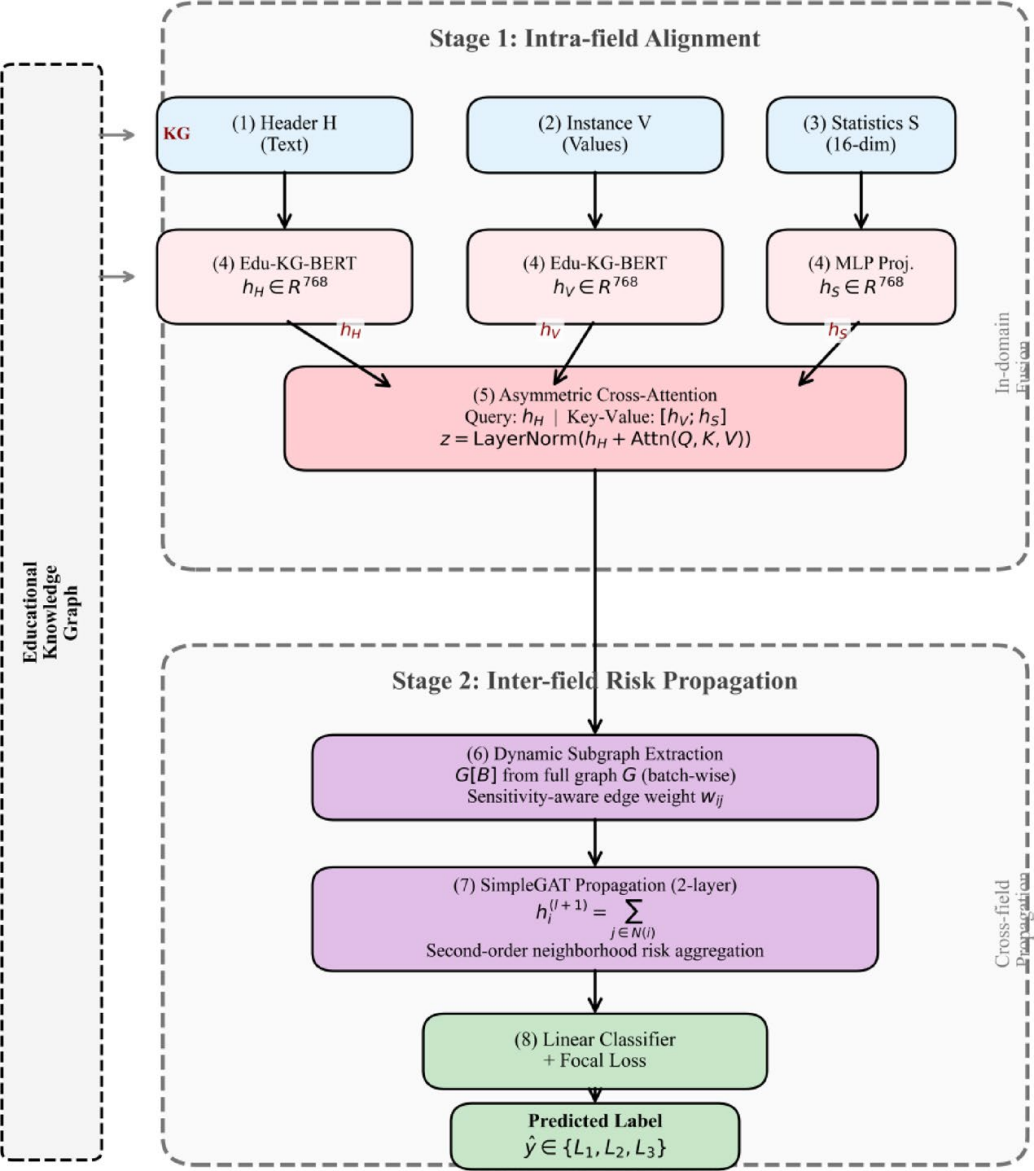
Overall model framework.

Addressing the challenges of semantic gaps, professional barriers, and small-sample overfitting in educational data, we propose SRP-Net, a knowledge-enhanced multi-channel semantic fusion network. The overall computational flow of this framework is shown in Fig. 1, mainly consisting of four core modules: educational domain knowledge injection encoder, metadata-instance multi-channel feature extraction, single-layer cross-attention based multi-modal fuser, and cost-sensitive optimization module for addressing class imbalance.

### Domain semantic injection based on knowledge graph (Edu-KG-BERT)

General-purpose pre-trained models (e.g., BERT-Base) often cannot understand the sensitive attributes of industry-specific terminology (e.g., “retired soldier program,” “three-assistance positions”) when processing specific vertical domain data. Studies show that directly injecting structured knowledge into pre-trained models is more efficient than relying on large-scale unlabeled corpora for Domain-Adaptive Pre-Training (DAPT) in data-scarce vertical domains^16^. Therefore, we constructed an educational sensitive data knowledge graph and implemented knowledge-enhanced domain adaptive pre-training.

#### Authority standard selection and knowledge source construction

To ensure the authority and coverage of the knowledge graph, we systematically sorted out three types of core policy standard documents:

National General Standards: Using the Information Security Technology—Personal Information Security Specification (GB/T 35,273) as the benchmark, defining the general classification logic of personal privacy data^34^.

Education Industry Standards: Selecting the Educational Management Information—Educational Management General Codes (JY/T 1001–2012), the Guidelines for Educational Data Classification and Grading, and relevant standard documents issued by provincial/municipal governments or universities, establishing hierarchical relationships for vertical business fields such as student status, grades, rewards, and punishments^35^.

Compliance Governance Manuals: Referencing real data asset catalogs from domestic universities, summarizing semantic mappings for large amounts of non-standard naming (e.g., “scholarship level” often mapped as “ax_dj”).

The reason for selecting the above standards is that they define the ground truth of educational sensitive data, providing rigid constraints for models from business terminology to security levels.

#### Knowledge triplet extraction and semantic transformation

Triple representation as shown in Table 2.

**Table 2:** Knowledge triplet examples.

Relation type	Example triplet	Semantic function
Business domain attribution	(ID Number, belongs to business domain, student basic information data sub-table)	Establishes subordination relationships between entities and business domains
Data type definition	(ID Number, data type is, C)	Clarifies field data formats (Character C/Numeric N, etc.)
Standard reference	(ID Number, references code or value domain, GB/T 11,643)	Associates with national/industry standard codes
Semantic explanation	(ID Number, defined or explained as, Citizen Identity Number)	Provides business semantic descriptions

For the parsing difficulties of PDF standard documents, including cross-page missing headers, mixed multi-format tables, and header residual noise, we adopted high-fault-tolerance parameter configurations and dynamic column mapping strategies, combined with data cleaning rules (field length filtering, null value elimination) to ensure extraction quality.

Knowledge graph construction pipeline. The Edu-KG was built through a reproducible semi-automatic pipeline: (1) Document parsing: pdfplumber and camelot-py extracted tables from PDF standards and compliance manuals, with dynamic column-mapping and regex-based field-length filtering to suppress cross-page missing headers, mixed-format tables, and header/footer noise; (2) Triplet generation: extracted entity-relation-entity fragments underwent manual verification to ensure that head and tail entities are core terms from educational data standards (e.g., “ID number,” “student status”), with relation types strictly limited to the four categories defined in Table 2—business-domain attribution, data-type definition, standard reference, and semantic explanation; (3) Quality cleaning: entities appearing fewer than twice were removed, synonymous entities were merged, and duplicates eliminated to yield a conflict-free, acyclic structure; (4) Scale statistics: Edu-KG is a schema-level domain ontology containing 2527 entities and 5404 triplets, with an average degree of 4.27 and a largest-connected-component coverage of 99.7%, indicating dense structural integrity for a vertical knowledge base.

#### Fact-judgment-based domain knowledge injection mechanism

Unlike general domain-adaptive pre-training (DAPT) that relies on massive unlabeled corpora, the educational sensitive data domain lacks sufficient open text for traditional Masked Language Modeling (MLM)^36^. Therefore, we propose a Supervised Knowledge Injection method for structured knowledge graphs, explicitly modeling logical relationships between entities to compensate for the domain blind spots of general BERT^16^.

(1) Positive Sample Semantic Encoding: Triplet Serialization and Structured Representation

Knowledge triplets in the knowledge graph exist in discrete symbolic form and cannot be directly processed by neural language models. Therefore, we designed a structured text sequence encoding strategy to transform triplets into continuous semantic representations conforming to BERT input specifications, drawing on the idea of unified encoding of knowledge embedding and language models^37^.

For a triplet (h,r,t) , where h is the head entity, r is the relation, and t is the tail entity, we use the following template for linearization:

2 $$SEQ(h,r,t){ = }[{\mathrm{CLS}}] \oplus h \oplus [{\mathrm{SEP}}] \oplus r \oplus [{\mathrm{SEP}}] \oplus t \oplus [{\mathrm{SEP}}]$$

(2) Domain-Aware Negative Sampling: Adversarial Sample Construction Under Relation Constraints

**Fig. 2:**
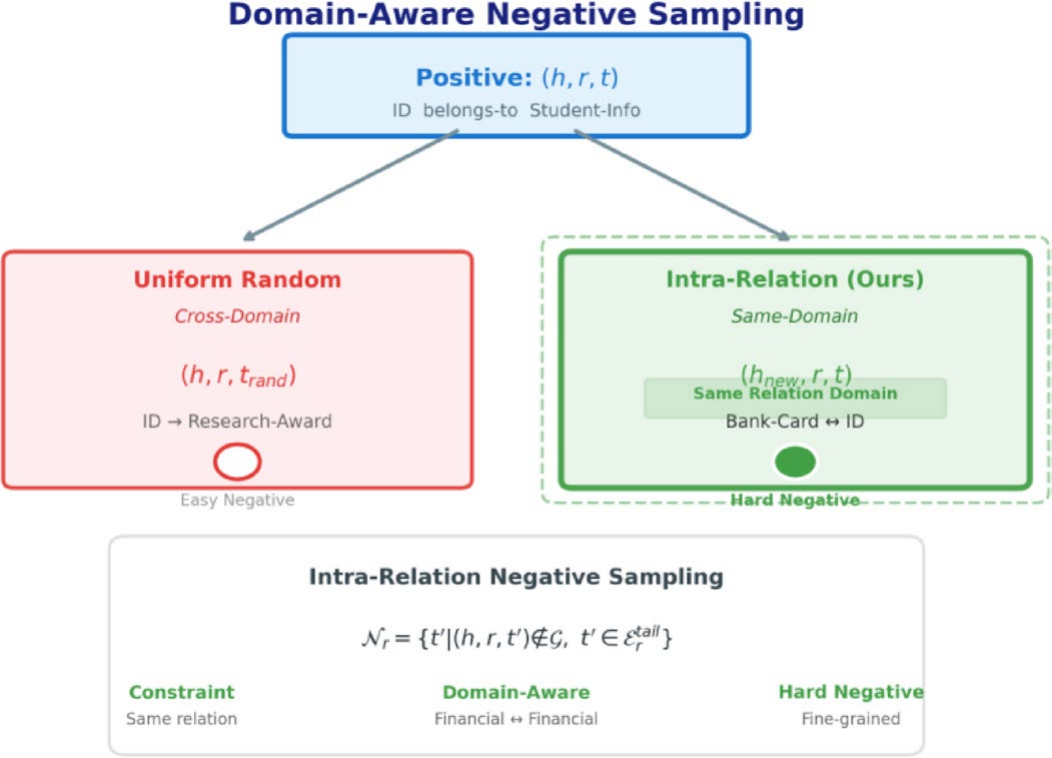
Negative sampling strategy diagram.

Existing negative sampling strategies^38^ introduce structure-aware mechanisms but still have semantic difference problems in cross-relation type sampling. We propose a relation-constrained negative sampling strategy, conducting entity replacement within the same relation type to generate hard negative samples with domain confusion, similar to the motivation of contrastive learning strategies in knowledge graph completion^39^.The specific strategy is shown in Fig. 2.

Same-Relation-Domain Negative Sampling. Given a positive sample triplet (h,r,t) , the negative sample construction space is:

3 $${\mathcal{N}}_{{{\mathrm{intra}}}} (h,r,t) = \left\{ {(h,r,t^{\prime } )t^{\prime } \in {\mathrm{E}}_{r}^{{{\mathrm{tail}}}} { \setminus }\{ t\} } \right\} \cup \left\{ {(h^{\prime } ,r,l)h^{\prime } \in {\mathrm{E}}_{r}^{{{\mathrm{head}}}} { \setminus }\{ h\} } \right\}$$

where ${\mathcal{E}}_{r}^{{{\mathrm{tail}}}}$ represents the set of all tail entities appearing for relation r in the knowledge graph.

(3) Knowledge-Enhanced Representation Learning: Triplet Factuality Classification Task

During the phase of injecting educational graph knowledge into the model, traditional domain adaptation often adopts unsupervised masked language modeling (MLM). However, MLM makes it difficult for models to explicitly learn directional logical relationships between entities^40^. Therefore, we reconstruct the graph knowledge injection process as a supervised Triplet Factuality Classification sequence classification task.

Specifically, we feed constructed positive samples (real triplets, y = 1 ) and hard negative samples (pseudo-triplets after entity replacement, y = 0 ) into the initialized bert-base-chinese encoder. The network extracts the [CLS] token vector at the sequence header and connects it to a randomly initialized binary classification linear layer (Linear Classification Head), computing cross-entropy loss for backpropagation:

4 $${\mathcal{L}}_{KG} = - \frac{1}{N}\sum\limits_{i = 1}^{N} {\left[ {y_{i} \log P(\hat{y}_{i} ) + (1 - y_{i} )\log (1 - P(\hat{y}_{i} ))} \right]}$$

Driven by this task, the model is forced to distinguish the collocation rationality of educational domain terminology in hidden space. After knowledge injection training (Epoch = 3), we directly discard the top-layer binary classification probe head and only persistently save the BERT encoder weights (Encoder Weights) that deeply integrate educational prior knowledge. This purified domain weight serves as the core semantic foundation for the subsequent SRP-Net multi-channel detection architecture.

### Multi-channel multi-dimensional feature extraction (dual-channel feature extraction)

Considering the extremely impoverished context of a single structured field, SRP-Net designs mutually independent but highly complementary feature extraction channels^13,14^:

Metadata Semantic Channel. For input field C , we extract its header name H . After tokenization, it is fed into Edu-KG-BERT. Since headers are usually extremely short, we hard-truncate the maximum length threshold to Nmax​ = 32 . The model extracts the specific token vector at position 0 as the high-dimensional semantic representation of the header:

5 $$h_{{{\mathrm{name}}}} = {\text{Edu - KG - BERT}}(H)_{{{\mathrm{CLS}}}} \in {\mathbb{R}}^{{d_{{{\mathrm{name}}}} }}$$

where d = 768.

Instance Value Evidence Channel. To bridge the semantic gap caused by non-standardized table header naming, the system goes deep into the database underlying layer for sampling. By extracting non-null values from this column and deduplicating based on string length to filter short noise, it selects Top-K (K≈8 ) long samples to concatenate into an instance snippet V . To ensure the balance between information coverage and computational efficiency, we truncate the maximum length to Vmax = 128 . Similarly, we extract its instance-level global representation:

6 $$h_{{{\mathrm{val}}}} = {\text{Edu - KG - BERT}}(V)_{{{\mathrm{CLS}}}} \in {\mathbb{R}}^{{d_{{{\mathrm{name}}}} }}$$

Statistical Fingerprint Projection. In addition to natural language, distribution features of numbers and symbols (e.g., length variance of ID cards, Chinese-English ratio, specific regular expression hit rates) are also key strong rules for discriminating high-sensitivity business data^41^. The system extracts 16-dimensional fixed statistical fingerprints with physical meaning S ∈ R^16^ from the instance stream and adopts Z-score standardization. Subsequently, through two layers of Multi-Layer Perceptron (MLP) combined with nonlinear activation functions, it projects them to high-dimensional continuous space, aligning semantic feature scales:

7 $$h_{{{\mathrm{stat}}}} = {\mathrm{ReLU}}(W_{2} \cdot {\mathrm{ReLU}}(W_{1} S + b_{1} ) + b_{2} ) \in {\mathbb{R}}^{64}$$

### Sensitive risk propagation network (SRP-Net)

Existing sensitive field recognition methods mostly adopt static feature concatenation or implicit graph convolution, making it difficult to explicitly model the dynamic semantic associations among “field name—value content—statistical fingerprint,” and full-graph propagation Graph Neural Networks (AGDN) suffer from over-smoothing and computational redundancy^6,12,21^. Therefore, this section proposes the Sensitive Risk Propagation Network (SRP-Net), achieving explicit alignment between modalities through asymmetric cross-attention and efficient knowledge graph risk propagation based on dynamic subgraph extraction strategies.

#### Asymmetric cross-attention fusion mechanism

Unlike traditional self-attention or gated fusion, SRP-Net adopts an asymmetric QKV structure (Fig. 1, Step (5); Algorithm 1, lines 10–12). Field names serve as Query, while concatenated values and statistics form Key-Value:

8 $${\mathbf{Q}} = h_{n} \in {\mathbb{R}}^{B \times 1 \times d} ,\quad {\mathbf{K}} = {\mathbf{V}} = \left[ {h_{v} \;;\;h_{s} } \right] \in {\mathbb{R}}^{B \times 2 \times d}$$

This forces the model to learn the directional dependency “header → content” rather than bidirectional entanglement. An 8-head scaled dot-product attention computes adaptive association strength, followed by residual connection and layer normalization:

9 $$\begin{gathered} {\mathrm{Attention}}({\mathbf{Q}},{\mathbf{K}},{\mathbf{V}}) = {\mathrm{Concat}}({\mathrm{head}}_{1} , \ldots ,{\mathrm{head}}_{s} ){\mathbf{W}}^{O} \hfill \\ {\mathrm{head}}_{i} = {\mathrm{Softmax}}\left( {\frac{{{\mathbf{QW}}_{i}^{Q} ({\mathbf{KW}}_{i}^{K} )}}{{\sqrt {d_{k} } }}} \right){\mathbf{VW}}_{i}^{V} \hfill \\ \end{gathered}$$

10 $${\mathbf{h}}_{fuse} = {\mathrm{LayerNorm}}({\mathbf{h}}_{{\mathrm{n}}} + {\mathbf{h}}_{{{\mathrm{attn}}}} )$$

This balances original semantic preservation with cross-modal context injection, solving the information truncation problem in gating mechanisms.

#### Risk propagation based on dynamic subgraphs

For the 2,942 field nodes in the training set,SRP-Net constructs a field correlation graph based on business domains and standard code relationships in the knowledge graph, and adopts a batch-wise subgraph extraction strategy to avoid the overhead and over-smoothing problems of full-graph computation^42^.

Edge weights are sensitivity-aware:

11 $$w_{ij} = 0.5 + 0.4 \times \left( {1 - \frac{{y_{i} - y_{j} }}{2}} \right)$$

where si, sj ∈ {0,1,2} . High-sensitivity nodes connect strongly (w≈0.9 ), while cross-level connections decay to 0.5. To avoid full-graph over-smoothing^12,42^, batch-wise induced subgraphs G[B] are extracted (Algorithm 1, lines 15–16):

12 $${\mathcal{E}}_{{{\mathrm{sub}}}} = \{ (i,j) \in {\mathcal{E}}i,j \in {\mathcal{B}}\}$$

A 2-layer SimpleGAT propagates risk via attention coefficients jointly determined by edge weight and semantic similarity (Algorithm 1, lines 17–22):

13 $$h_{j}^{(l + 1)} = {\mathrm{LayerNorm}}\left( {h_{j}^{(l)} + \sum\limits_{j \in N(i)} {\alpha_{ij} } W^{(l)} h_{j}^{(l)} } \right)$$

achieving second-order neighborhood aggregation without full-graph computation overhead.

It should be noted that the knowledge graph G is constructed from the training-set field vocabulary (|V|= 2,942). Consequently, during inference on unseen schemas (e.g., cross-institutional zero-shot migration), test fields that do not appear in the training vocabulary cannot be indexed into G, causing the GNN layer to degenerate into isolated-node encoders. This limitation is inherent to static graph construction and will be empirically analyzed in Sect. “Cross-institutional zero-shot evaluation”.

**Algorithm 1:**
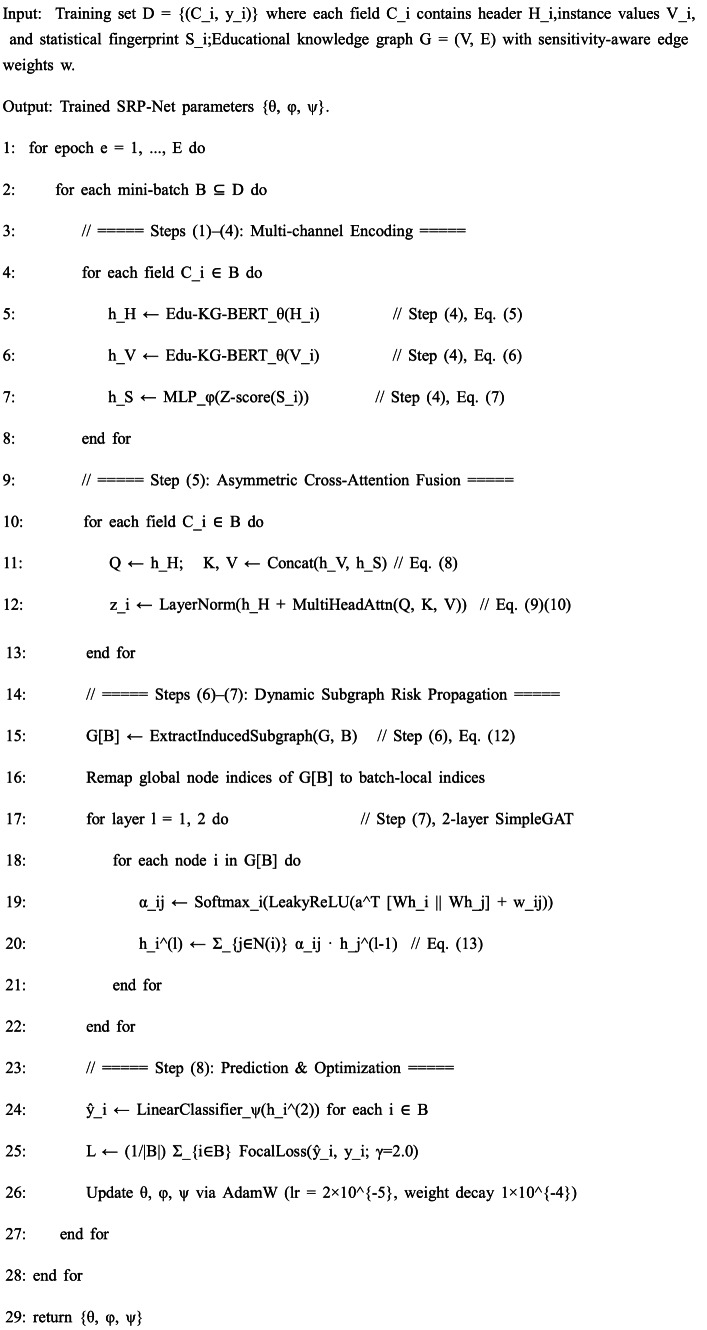
SRP-Net training and inference procedure.

## Experimental evaluation

### Implementation details and metrics

Experiments are implemented based on PyTorch 2.0.1. All deep models load Edu-KG-BERT weights, freezing the first 4 layers of BERT and opening higher layers for fine-tuning. Input sequence maximum lengths: field name 32, instance value 128; statistical fingerprints are projected to 16 dimensions after Z-score standardization. Training adopts AdamW optimizer (initial learning rate 2 × 10 − 5 , weight decay 1 × 10 − 4 ), linear Warmup accounting for 10%, Batch Size of 8 (gradient accumulation steps 2, equivalent to 16), training for 10 epochs. Category-weighted Focal Loss (γ = 2.0) is used to alleviate class imbalance. The main experiment fixes random seed 42. Multi-seed stability tests for fusion-strategy comparison (Table 6) employ five independent seeds (42, 123, 456, 789, 2024). CuDNN non-deterministic optimization is disabled throughout to ensure reproducibility. Evaluation metrics adopt Macro-F1 (core metric), Accuracy (auxiliary), and trainable parameter count (model complexity).

### Main Results Analysis

To compare the performance of different models on the educational sensitive dataset, we selected typical models from traditional machine learning and deep learning as comparisons. Specific experimental results are shown in Table 3.

**Table 3:** Performance comparison of different models on Edu-sens dataset.

Method category	Baseline model	Macro-F1	Accuracy	Parameters
Traditional ML	RandomForest (StatOnly)	0.4558	0.7000	–
	LightGBM (StatOnly)	0.4625	0.7580	–
	XGBoost(StatOnly)	0.4683	0.7621	–
General deep learning	PureMLP (StatOnly)	0.3220	0.5821	0.5 M
	BERT-Base(NameOnly)	0.8214	0.9358	102 M
Multi-modal fusion baselines	SimpleConcat(Name + Value + Stat)	0.8564	0.9412	74.3 M
	Double-gated fusion	0.8697	0.9538	77.6 M
Our method	SRP-Net	0.8779	0.9558	77.7 M

As shown in Table 3, SRP-Net achieves 0.8779 Macro-F1 and 95.58% accuracy on the Edu-Sens test set, significantly outperforming all baseline models. Through systematic comparison of various metrics, we distill key insights from the following four dimensions:

(1) Semantic Gap: Pure statistical methods face insurmountable performance ceilings.

Traditional ML models (RF/LightGBM/XGBoost) plateau at 0.4558–0.4683 Macro-F1, confirming that statistical fingerprints alone cannot map non-standard naming to sensitivity levels, causing dark data to be missed.

(2) Domain Gap: General pre-trained models have vertical business blind spots.

BERT-Base (NameOnly) achieves 0.8214 F1, 5.38% below SRP-Net, systematically misjudging education-specific terms (e.g., “poverty registration”). KG-BERT establishes rigid constraints from business terminology to security levels, validating the necessity of domain knowledge injection.

(3) Multi-modal Complementarity: Instance value channels crack header naming failure crises.

SimpleConcat (0.8564) improves over BERT-Base by 3.50%, proving instance values compensate for header blind spots. However, flattening three modalities forces implicit alignment learning, under-converging on 3,000 low-resource samples.

(4) Alignment through Simplicity: Explicit single-layer attention outperforms implicit deep entanglement.

Double-Gated Fusion (0.8697) underperforms SRP-Net (0.8779) despite equivalent parameters, as pre-fusion Value ↔ Stat entanglement causes gradient competition. Single-CrossAttn explicitly models “Name → Value/Stat” dependency, achieving better decoupling and discriminative retention.

### Ablation study and architecture analysis

To verify the independent contributions of core modules in the SRP-Net framework and explore optimal multi-modal fusion strategies, we designed systematic ablation experiments. All experiments were conducted under strictly fair conditions: sharing the same random seed, same training configuration (10 epochs, freezing first 4 BERT layers), and same evaluation protocol to ensure comparability of results.

#### Multi-modal component ablation

By progressively decoupling domain pre-training knowledge (KG), semantic channels (Value), and statistical features (Stat), we evaluated the marginal contributions of each modality to the sensitive field detection task. Results are shown in Table 4.

**Table 4:** Component ablation experiments.

Ablation variant	KG	Value	Stat	Accuracy	Macro-F1	Parameters	ΔF1
CrossAttn-only baseline	✓	✓	✓	0.9554	0.8473	76.63 M	–
w/oKG	✗	✓	✓	0.9538	0.8109	74.33 M	− 3.64%
w/oValue	✓	✗	✓	0.9442	0.8130	73.92 M	− 3.43%
w/oStat	✓	✓	✗	0.9468	0.8347	74.31 M	− 1.26%
w/oKG&Value	✗	✗	✓	0.7000	0.2915	–	− 55.58%

**Fig. 3:**
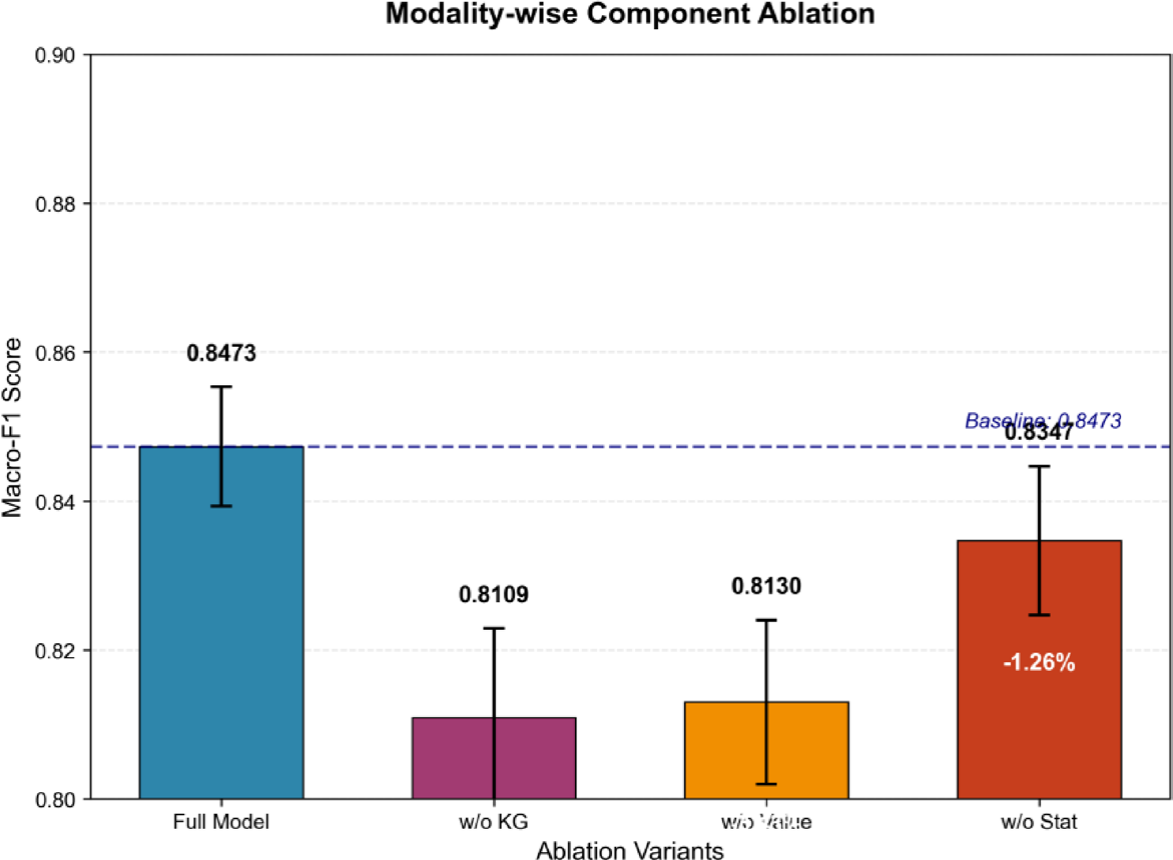
Modality-wise component ablation on the CrossAttn-Only baseline.

From Fig. 3 and Table 4, it can be concluded that, the CrossAttn-Only baseline achieves 0.9554 accuracy and 0.8473 Macro-F1 with 76.63 M parameters.

Removing KG drops F1 by 3.64% (0.8109), removing Value drops by 3.43% (0.8130), and removing Stat drops by only 1.26% (0.8347). The catastrophic collapse of w/o KG&Value (0.2915, − 55.58%) confirms that deep learning underperforms tree-based models on low-dimensional tabular data unless semantic modalities (KG + Value) break the performance ceiling^8^. This reveals strong nonlinear complementarity between KG-provided “entity definition” and Value-provided “contextual positioning.”

#### Fusion module ablation

To verify the independent contributions of graph-based risk propagation components built upon the cross-attention fusion mechanism, we conducted modular stripping experiments on the fusion layer while keeping the Edu-KG-BERT encoder and input configuration completely consistent. Fixing seed to seed = 42, we quantitatively evaluated the marginal contribution of each component to model discrimination capability. Experimental results are shown in Table 5.

**Table 5:** Fusion module ablation experiments.

Ablation variant	Macro-F1	Parameters	ΔF1
Full-model	0.8779	77.71 M	–
w/oGNN	0.8473	76.63 M	− 3.06%
w/oStandard	0.8480	77.71 M	− 2.99%
w/oWeight	0.8603	77.71 M	− 1.76%
w/oDomain	0.8670	77.71 M	− 1.09%
Random graph	0.8727	76.53 M	− 0.52%

**Fig. 4:**
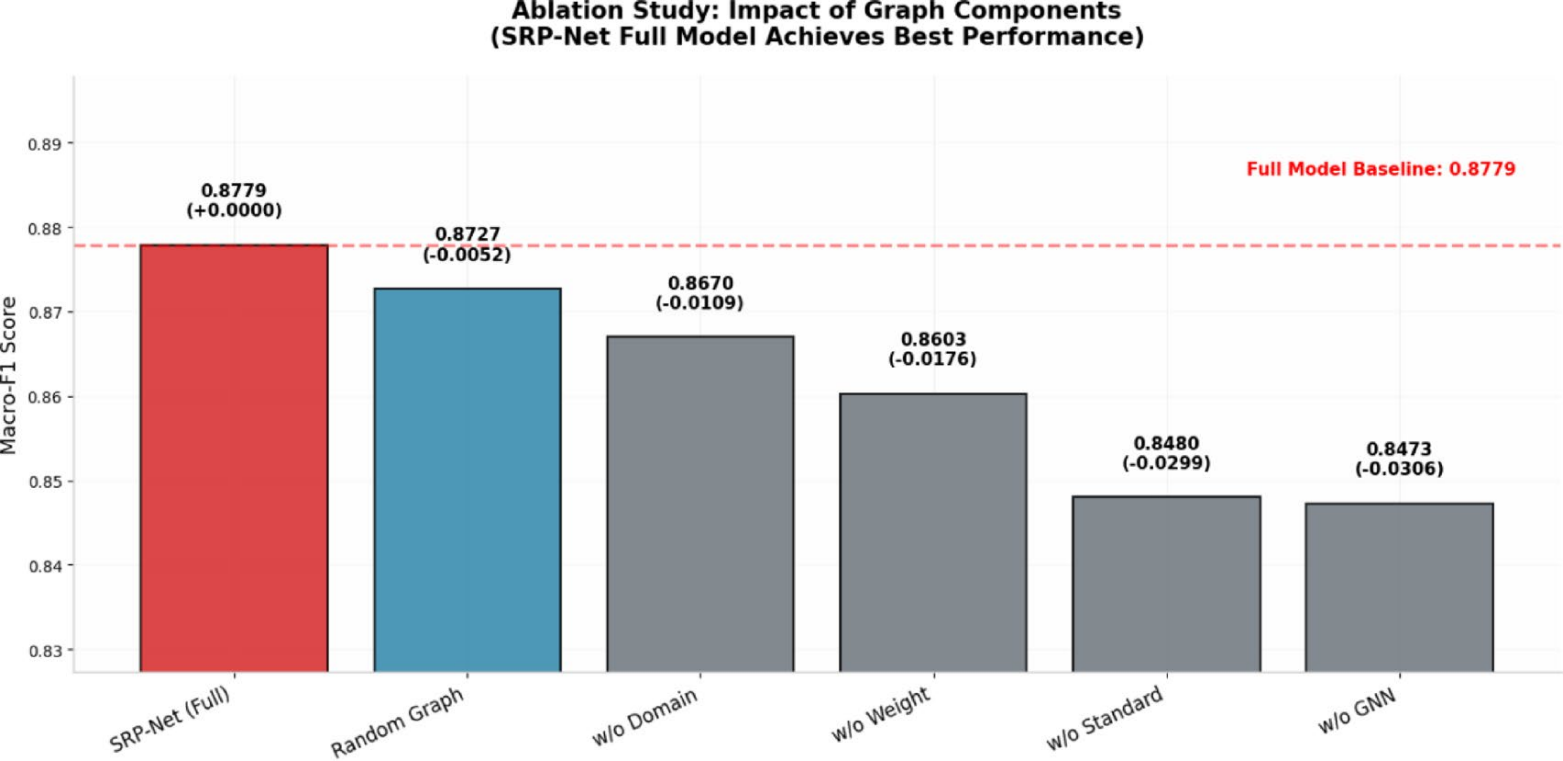
Comparison of fusion module ablation.

Ablation results (Table 5 and Fig. 4) show that removing the GNN layer causes significant performance degradation of 3.06% (0.8779 → 0.8473), indicating that learnable risk propagation mechanisms are core to performance improvement rather than just adding parameters (reducing 1.08 M). Missing standard-code edges causes greater loss (− 2.99%) than business-domain edges (− 1.09%), revealing that technical references carry stronger sensitivity signals. Uniform weights (w/o Weight) drop F1 by 1.76%, validating sensitivity-aware weighting. The Random Graph variant (− 0.52%) outperforms w/o Domain and w/o Weight individually, indicating GNN structural fault tolerance, while KG-guided connections provide additional semantic constraints.

**Fig. 5:**
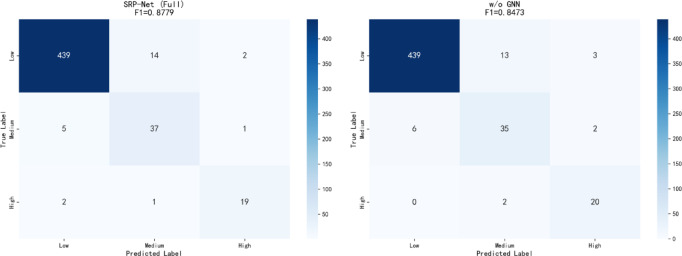
SRP-Net confusion matrix diagram.

Figure 5 confirms that GNN propagation improves high-sensitivity precision (80.0% → 86.4%) and medium-sensitivity recall (35/43 → 37/43), while both variants rarely exhibit cross-level errors (Low ↔ High), indicating that cross-attention establishes basic alignment and GNN further refines boundary precision.

#### Architecture exploration of fusion strategies

To verify the optimal topological structure of the multi-modal fusion layer, we further compared multiple architecture variants from simple concatenation to complex attention mechanisms. All variants were based on the same KG-BERT encoder and input configuration, modifying only the fusion layer structure, and were conducted with 5 independent repeated experiments under different random seeds. Results are shown in Table 6.

**Table 6:** Statistics of different fusion methods.

Fusion strategy	Trainable parameters (M)	Macro-F1 score	Accuracy	Time_per_Epoch(s)
Single-CrossAttn	76.63	0.8473 ± 0.0086	0.9554 ± 0.0045	41.33
Double-Gated	77.61	0.8697 ± 0.0127	0.9538 ± 0.0021	40.21
Hierarchical-BiAttn	79.98	0.8660 ± 0.0149	0.9538 ± 0.0047	41.40
Concat-baseline	74.33	0.8654 ± 0.0095	0.9538 ± 0.0032	40.82
AGDN-Res	77.71	0.8638 ± 0.0093	0.9515 ± 0.0028	41.37
SRP-Net	77.71	0.8779 ± 0.0114	0.9558 ± 0.0032	168.6

Table 6 confirms that complex fusion (Hierarchical-BiAttn 0.8660 ± 0.0149, Double-Gated 0.8697 ± 0.0127) triggers over-parameterization and instability in low-resource scenarios. AGDN-Res (0.8638) underperforms SRP-Net by 1.41% despite identical parameters, proving dynamic subgraph extraction avoids full-graph over-smoothing. The 3.06% gain over Single-CrossAttn aligns with Table 5, jointly validating GNN’s contribution under both single-seed and multi-seed protocols.

#### Backbone generalization

To verify architecture-agnosticism, we instantiate the full SRP-Net architecture with three Chinese PLM backbones (BERT-Base, RoBERTa-wwm, MacBERT), replacing only the KG-BERT encoder. All variants share identical training configurations (seed = 42, 10 epochs, freeze first 4 layers, Focal Loss).

As shown in Table 7, RoBERTa-wwm and MacBERT outperform the vanilla BERT-Base backbone by 2.24 and 2.22 percentage points, respectively, confirming benefits from improved pre-training. Yet even the strongest general-purpose backbone (RoBERTa-wwm, 0.8639) underperforms our domain-adapted KG-BERT (0.8779). This gap validates that while SRP-Net’s fusion and propagation modules transfer across encoders, vertical domain adaptation remains indispensable for conquering educational terminology blind spots.

**Table 7:** Generalization across PLM backbones (Edu-Sens, single run, seed = 42).

Backbone	Macro-F1	Explanation
BERT-Base	0.8415	Baseline base, no domain knowledge
RoBERTa-wwm	0.8639	Full word mask pre training
MacBERT	0.8637	Correcting pre training—fine-tuning inconsistency
KG-BERT	0.8779	Domain knowledge injection

All rows report the full SRP-Net architecture (asymmetric cross-attention + dynamic subgraph + statistical fingerprints) with only the encoder backbone varied. The row BERT-Base (Full Arch.) (0.8415) is distinct from the NameOnly baseline in Table 3 (0.8214), which omits the Value, Stat, and GNN modules.

### Case study and interpretability

To verify SRP-Net’s robustness and error correction capabilities on real heterogeneous educational data, we conducted empirical analysis from two dimensions: the effectiveness of knowledge graph risk propagation mechanisms and the error correction effects of multi-channel instance injection.

**Fig. 6:**
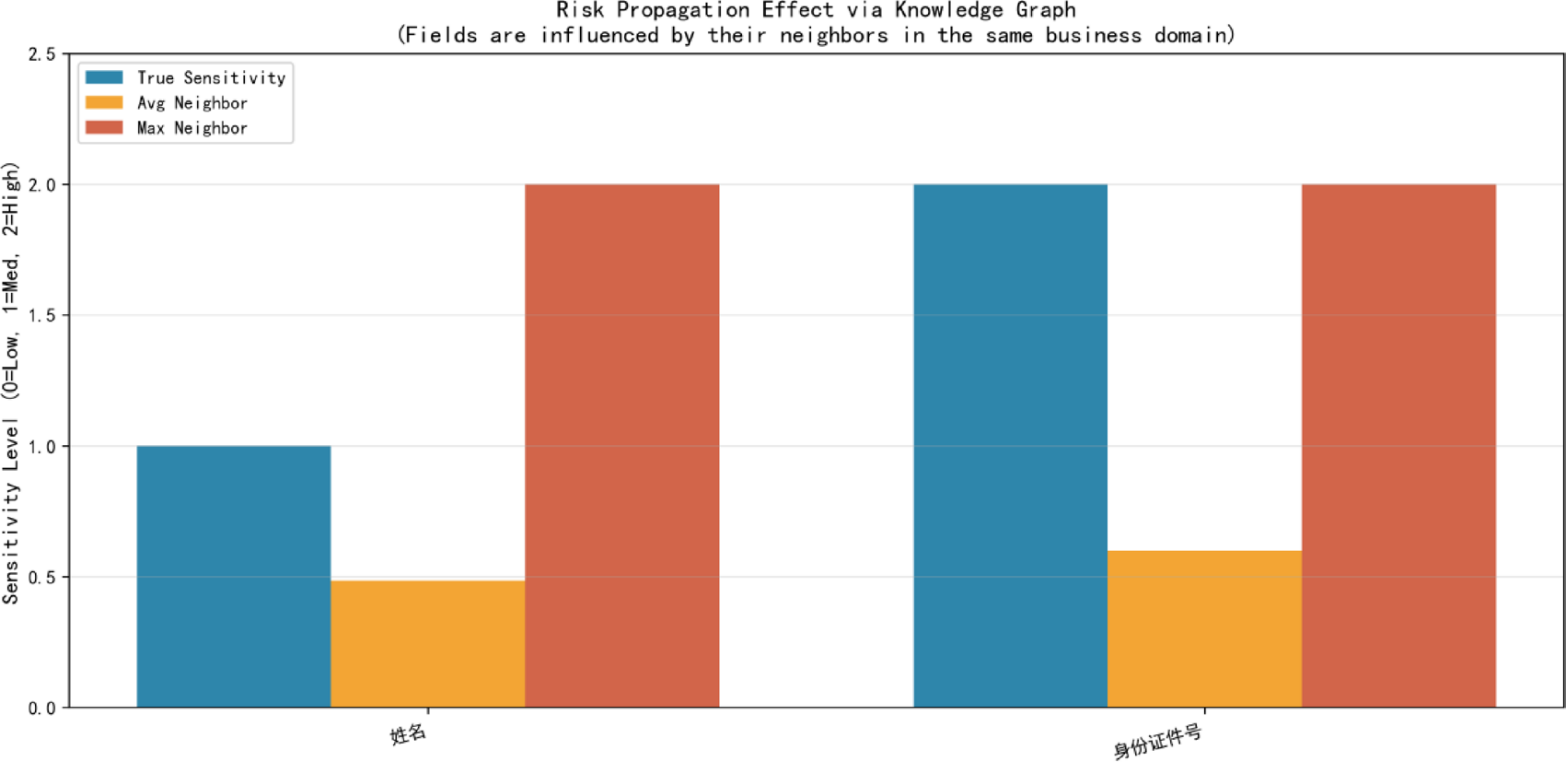
Sensitivity propagation diagram.

Figure 6 and Table 8 demonstrate SRP-Net’s error-correction synergy. In the “Student Basic Information” domain, “Name” (True Sensitivity = 1.0) neighbors high-sensitivity fields (Max Neighbor = 2.0); second-order GNN propagation prevents misclassification as public. Conversely, for header-semantic misleading, single-modal baselines misclassify “Check Number” as high-sensitivity due to the word “number,” while SRP-Net’s asymmetric cross-attention penetrates naming noise via underlying instance values (numeric codes vs. institution names), correcting all five adversarial cases.

**Table 8:** Case study on correcting header misleading.

Field name (header)	Sampled instance value snippet	True label	Single-modal baseline (NameOnly)	Our model (Name + Value)
Household registration processing status code	437,968, 876,416, 306,227…	L1 (Public)	L3 (High)	L1 (Public)
Check number	235,703, 265,967, 919,475…	L1 (Public)	L3 (High)	L1 (Public)
current organization ID	Harbin Medical University, Zhengzhou University of Technology…	L2 (Medium)	L1 (Public)	L2 (Medium)
Enrollment method	In Progress, Archived, Pending Improvement…	L1 (Public)	L2 (Medium)	L1 (Public)
Province/city code	231,699, 641,527, 667,851…	L1 (Public)	L2 (Medium)	L1 (Public)

This synergy of within-field alignment and cross-field propagation resolves cross-modal semantic gaps in low-quality, heterogeneous data. Detailed attention-weight distributions cases are provided in Supplementary Material Table 1 and Fig. 1.

### Cross-institutional zero-shot evaluation

To assess generalization under extreme domain shift, we evaluate zero-shot transfer across institutions (University A → B non-overlapping fields, 1,226 test samples, 438 overlapping fields excluded). Each configuration is run 5 times with independent seeds (42, 123, 456, 789, 2024); results are reported as mean ± std in Table 9.

**Table 9:** Cross-institutional evaluation (A → B, n = 5).

Model	Accuracy	Precision	Recall	Macro-F1
SRP-Net	0.9307** ± **0.0036	0.8428** ± **0.0254	0.7695** ± **0.0243	0.7992** ± **0.0079
BERT + Value	0.9377** ± **0.0017	0.8678** ± **0.0227	0.7723** ± **0.0199	0.8127** ± **0.0098
SRP-Net(w/o GNN)	0.9346** ± **0.0013	0.8650** ± **0.0194	0.7596** ± **0.0152	0.8044** ± **0.0082

All variants converge to ~ 80% Macro-F1 with overlapping confidence intervals, indicating that field-name semantics constitute the dominant transferable signal under severe domain shift, while architectural differences are muted by cross-domain instance-value noise.

Two observations are noteworthy. (1) GNN degeneration: Full SRP-Net trails w/o-GNN (− 0.52%), confirming that out-of-vocabulary test fields reduce the GNN to isolated-node encoders (Section “Risk propagation based on dynamic subgraphs”). (2) Distribution-shift vulnerability: BERT + Value marginally leads because source-domain value patterns do not generalize; SRP-Net’s forced name → value dependency propagates noisy statistics, whereas simple concatenation autonomously suppresses the corrupted channel.

These results delineate SRP-Net’s applicability boundary: the proposed mechanisms excel in-domain but require domain-adaptive normalization or dynamic graph reconstruction for robust cross-institutional deployment.

## Conclusion

This paper proposes SRP-Net, a dual-layer architecture of “within-field explicit alignment and cross-field risk propagation” for low-resource educational sensitive data detection. Asymmetric cross-attention models the directional dependency “header → content,” overcoming semantic absence from heterogeneous naming. Dynamic subgraph extraction achieves second-order neighborhood risk propagation without full-graph over-smoothing. Experiments on the open-source Edu-Sens benchmark (0.8779 Macro-F1) validate that explicit alignment outperforms implicit deep entanglement (+ 0.82% over Double-Gated, + 1.41% over AGDN-Res) and that structured knowledge injection is indispensable (+ 3.64 percentage points over the BERT-Base backbone in the full architecture, Table 7). Future work will explore model lightweighting for edge deployment and federated multi-institutional reasoning under privacy constraints.

## Supplementary Information

Below is the link to the electronic supplementary material.

Supplementary Material 1

## Data Availability

The Edu-Sens benchmark dataset is publicly available at https://www.kaggle.com/datasets/baiputao/edu-sens. In compliance with the Data Security Law of the People’s Republic of China and privacy protection protocols, this dataset contains authentic table schemas (3,462 field headers) collected from real educational management systems, combined with synthetic instance values generated through LLM and rule-based engines to ensure no genuine personal information is disclosed. The source code for SRP-Net and the educational knowledge graph (Edu-KG) are available from the corresponding author upon reasonable request. The Edu-KG contains structured metadata—including business-domain classifications, standard code mappings, and sensitivity-level annotations—extracted from internal data-asset catalogs and compliance governance manuals of partner universities. To protect the internal business logic and data-governance protocols of these institutions, the Edu-KG triplet files, entity vocabulary, and construction scripts are not released for direct download alongside the dataset. Researchers with reproducibility needs may submit a usage request to the corresponding author (mhzheng3@163.com), stating the research purpose and intended data-use scope; upon review, we will provide a sanitized version of the knowledge graph files and construction scripts, thereby balancing reproducibility with institutional data-security compliance.
