# Supplementary material for: SRP-Net: sensitive risk propagation network with asymmetric cross-attention for educational data
Source: Sci Rep. 2026 Jun 4;16:25627. doi: 10.1038/s41598-026-53482-8 (PMC13478154; doi:10.1038/s41598-026-53482-8)
Supplement: Supplementary file 1 [file 41598_2026_53482_MOESM1_ESM.docx]

Supplementary Material

| **Table 1: Cross-Institutional Misclassification Case Analysis** | | | | |
| --- | --- | --- | --- | --- |
| **Field Name** | **True Label** | **SRP-Net (Wrong)** | **BERT+Value (Correct)** | **Attention Distribution** |
| Candidate  Communication Address | High | Public | High | Value: 0.698 |
| Independent Legal Entity | Medium | Public | Medium | Value: 0.653 |
| Spouse Personnel Type | Public | Medium | Public | Stat: 0.641 |
| Student Origin Location | Medium | Public | Medium | Value: 0.652 |
| Administrator | Medium | Public | Medium | Balanced: 0.449 / 0.551 |

****Observation.**** In 4 out of 5 cases, SRP-Net assigns dominant attention to the Value channel (>0.65), forcing the name representation to align with corrupted instance patterns. BERT+Value, unconstrained by the asymmetric Query-Key-Value structure, autonomously suppresses the noisy channel and relies on header semantics for correct judgment. The case "配偶人员类型" further reveals that when statistical features (Stat) dominate (0.641), SRP-Net can also be misled by distributional fingerprints that do not generalize across institutions. These cases empirically corroborate the trade-off hypothesis in Section 5.5: explicit alignment improves in-domain robustness but sacrifices adaptability under distribution shift.

**Figure 1. Attention Weight Graph**

**Table 2: Time Cost Table**

| Model | Seed | Best_Epoch | Time_per_Epoch(s) |
| --- | --- | --- | --- |
| Concat-Baseline | 42 | 10 | 44.317114520072934 |
| Concat-Baseline | 123 | 6 | 40.22739531993866 |
| Concat-Baseline | 456 | 10 | 40.18107666969299 |
| Concat-Baseline | 789 | 9 | 40.19026873111725 |
| Concat-Baseline | 2024 | 9 | 40.19736118316651 |
| AGDN-Res | 1042 | 10 | 41.37602953910827 |
| AGDN-Res | 1123 | 7 | 41.037446928024295 |
| AGDN-Res | 1456 | 10 | 41.04533724784851 |
| AGDN-Res | 1789 | 10 | 40.99975650310516 |
| AGDN-Res | 3024 | 6 | 41.01827940940857 |
| Double-Gated | 2042 | 9 | 41.28850305080414 |
| Double-Gated | 2123 | 7 | 40.926730728149415 |
| Double-Gated | 2456 | 7 | 40.87405550479889 |
| Double-Gated | 2789 | 7 | 40.88822190761566 |
| Double-Gated | 4024 | 7 | 40.85084705352783 |
| Single-CrossAttn | 3042 | 10 | 41.1432067155838 |
| Single-CrossAttn | 3123 | 8 | 41.22894148826599 |
| Single-CrossAttn | 3456 | 10 | 41.194383430480954 |
| Single-CrossAttn | 3789 | 7 | 41.14912033081055 |
| Single-CrossAttn | 5024 | 8 | 41.1417474269867 |
| Hierarchical-BiAttn | 5042 | 9 | 41.371047377586365 |
| Hierarchical-BiAttn | 5123 | 9 | 41.450520038604736 |
| Hierarchical-BiAttn | 5456 | 10 | 41.388255906105044 |
| Hierarchical-BiAttn | 5789 | 7 | 41.42059955596924 |
| Hierarchical-BiAttn | 7024 | 10 | 41.42900698184967 |
| SRP-Net | 42 | 10 | 165.8890323234834 |
| SRP-Net | 123 | 10 | 172.5418892356713 |
| SRP-Net | 456 | 8 | 165.8822314566314 |
| SRP-Net | 789 | 9 | 168.1278901345901 |
| SRP-Net | 2024 | 8 | 170.6789123098361 |

| ****Table 3. Notation and Symbol Definitions**** | | |
| --- | --- | --- |
| **Symbol** | **Dimension / Type** | **Description** |
| C | — | A database field to be detected |
| H | R^d^ | Header metadata embedding (token 0 of Edu-KG-BERT output) |
| V | R^d^ | Instance value sequence embedding |
| S | R^16^ | Statistical fingerprint vector |
| d | 768 | Hidden dimension of BERT encoder |
| B | — | Mini-batch of fields |
| Q,K,V | R^d^ | Query, Key, Value in asymmetric cross-attention |
| z_i_ | R^d^ | Fused representation of field i after cross-attention |
| G=(V,E) | — | Sensitivity-aware knowledge graph |
| w_ij_ | scalar | Edge weight between nodes i and j |
| α_ij_ | scalar | GNN attention coefficient |
| h_i_^(l)^ | R^d^ | Hidden representation of node i at layer l |
| N(i) | — | Neighborhood set of node i in subgraph |
| y | {L_1_,L_2_,L_3_} | Sensitivity label (Public / Medium / High) |
